# Very Large Pores Mesoporous Silica as New Candidate for Delivery of Big Therapeutics Molecules, Such as Pharmaceutical Peptides

**DOI:** 10.3390/ma16114151

**Published:** 2023-06-02

**Authors:** Debora Carrozza, Gianluca Malavasi, Erika Ferrari

**Affiliations:** Department of Chemical and Geological Sciences, University of Modena and Reggio Emilia, Via G. Campi 103, 41125 Modena, Italy; debora.carrozza@unimore.it (D.C.); erika.ferrari@unimore.it (E.F.)

**Keywords:** large pores’ mesoporous silica, Nisin, pharmaceutical peptides, porous biomaterials

## Abstract

The synthesis of a scaffold that can accommodate big molecules with a pharmaceutical role is important to shield them and maintain their biological activity. In this field, silica particles with large pores (LPMS) are innovative supports. Large pores allow for the loading of bioactive molecules inside the structure and contemporarily their stabilization and protection. These purposes cannot be achieved using classical mesoporous silica (MS, pore size 2–5 nm), because their pores are not big enough and pore blocking occurs. LPMSs with different porous structures are synthesized starting from an acidic water solution of tetraethyl orthosilicate reacting with pore agents (Pluronic^®^ F127 and mesitylene), performing hydrothermal and microwave-assisted reactions. Time and surfactant optimization were performed. Loading tests were conducted using Nisin as a reference molecule (polycyclic antibacterial peptide, with dimensions of 4–6 nm); UV-Vis analyses on loading solutions were performed. For LPMSs, a significantly higher loading efficiency (LE%) was registered. Other analyses (Elemental Analysis, Thermogravimetric Analysis and UV-Vis) confirmed the presence of Nisin in all the structures and its stability when loaded on them. LPMSs showed a lower decrease in specific surface area if compared to MS; in terms of the difference in LE% between samples, it is explained considering the filling of pores for LPMSs, a phenomenon that is not allowed for MSs. Release studies in simulated body fluid highlight, only for LPMSs, a controlled release, considering the longer time scale of release. Scanning Electron Microscopy images acquired before and after release tests shows the LPMSs’ maintenance of the structure, demonstrating strength and mechanical resistance of structures. In conclusion, LPMSs were synthesized, performing time and surfactant optimization. LPMSs showed better loading and releasing properties with respect to classical MS. All collected data confirm a pore blocking for MS and an in-pore loading for LPMS.

## 1. Introduction

In the actual scenery, immunoresistance against viruses, bacteria and fungi plays a key role in the current scientific research. Antimicrobial peptides (AMPs) have attracted great attention as a potential substituent for conventional antibiotics [1,2]. This appends previous research because they have recently been targeted as novel antimicrobial agents with the potential to treat multiple drug-resistant infections [2,3]. For this reason, peptides with antimicrobial and anticancer activities could be a new pathway to follow [4,5,6].

The administration and the in situ delivery and release of peptides cannot be easily performed, because of their denaturation caused by peptidase present in biological fluids. In fact, AMPs can hardly survive in the environment of infected tissue and have poor physiochemical stability with a short circulating plasma half-life [1,7,8]. So, increasing the bioavailability or stability of peptide drugs through delivery or formulation is the key to the clinical applications of peptides [1,3,9].

Following this philosophy, the synthesis of a scaffold that can accommodate these big molecules is important to shield them and maintain their biological activity [10]. Their conjugation with various classes of materials and their delivery via carrier systems are strategies being used to enhance their therapeutic efficacy.

In this field, silica particles with large pores (comparable to the peptides dimensions) are innovative and can provide interesting support [10]. In fact, large pores could allow for the loading of the bioactive molecule inside the structure and contemporarily allow for their stabilization and protection from peptidase [11]. These purposes cannot be achieved using classical mesoporous silica (MS, with pore size of 2–5 nm) [12], because their pores are not big enough to enable the insertion inside them, and only external loading can be performed [13]. Pores’ size is an important aspect because it has been demonstrated [10] that the dependence of in vitro adsorption/release of AMPs is a function of the mesopores’ size. Results obtained prove that the mesopores’ size critically influences the encapsulation of AMPs, and at low concentrations, encapsulation can be achieved only for the structures in which mesopores’ size are in the range of peptide dimensions.

Mesoporous silica can be synthesized starting from a solution containing the following: tetraethyl orthosilicate (TEOS), which is a silica structure precursor, a pore agent that can be tuned (in terms of quantity and type) according to pores’ dimensions that you want to obtain and an adjuvant of pore agent that helps to obtain larger pores [14,15,16].

Furthermore, with the idea of obtaining smart materials, silica particles can be loaded with the pharmacologically bioactive molecule, and then the particles can be covered with an intelligent coating (sensible to variation of environmental potential, that occurs near the tumoral environment) that detaches from the surface and permits the in situ release of the drug [17,18,19].

The aim of this work was to find a new synthesis pathway for obtaining LPMS particles, with a high pores’ dimension range; then, we performed the optimization of the synthesis in terms of surfactant used [20] and reaction time. Later, LPMSs were used as support for loading a peptide, and simultaneously, loadings were also performed into classical MS to have a comparison sample. Additionally, releases were performed for both LPMSs and MS. All results collected (SSA, TGA-DTA curves, UV-VIS analysis and EA) demonstrate a higher encapsulation of the peptide inside LPMSs and a controlled and longer release. SEM-FEG images acquired for LPMSs after release tests confirm the improvement of mechanical resistance and strength of the structure. Not so good results were obtained for MS, demonstrating that LPMSs are better candidates to be used as drug delivery systems of big therapeutic molecules.

## 2. Materials and Methods

### 2.1. General Procedures

All the chemicals and solvents were purchased with the highest purity grade available and used without further purification unless otherwise specified. To synthetized LPMSs, hydrothermal treatments were performed with microwaves (MWs), using FlexiWAVE (Milestone S.r.l., Sorisole, Italy) 230 V.

### 2.2. Synthesis of LPMSs

The synthesis of LPMS was adapted based on earlier studies [16,20]. In general, TEOS is used as silica structure precursor, where Pluronic surfactant is the pore agent and TMB is the adjuvant of surfactant.

In this work, to synthesize LPMSs, we started from an acidic water solution (1.7% *w*/*w* HCl) containing the following: tetraethyl orthosilicate (TEOS) (98%, Merck, Darmstadt, Germany), Pluronic^®^ surfactant P123 or F127 (>99.9%, Merck, Darmstadt, Germany) and 1,2,3-Trimethylbenzene (TMB) (100%, Merck, Darmstadt, Germany). The solution was hydrothermally treated with MWs, starting with a thermal treatment at 80 °C for 6 h and then followed by a second thermal treatment at 160 °C for 12 h. MWs’ power was dosed by the instrument based on the solution temperature.

Different molar ratios between TEOS and Pluronic were studied in addition to different reaction times. Furthermore, syntheses with and without TMB were performed to evaluate the effect of TMB on pores’ dimensions.

All syntheses conducted are reported in Table 1. Samples were named as LPMS_n_TMB_yh, in which n is the chronological number of the synthesis, TMB indicates the presence or not of the adjuvant and y is the number of hours if it is different from 18 h (in these cases, the ratio between the times of treatment at 80 °C and 160 °C was maintained).

After thermal treatment, synthesis solutions were filtered, the collected solids were dried overnight at 60 °C and then they were calcined at 2 °C/min up to 700 °C for 3 h, under an air atmosphere to remove the surfactant as well as to stabilize the resultant mesoporous glasses. After quenching in air, the LPMSs were gently milled in an agate mortar and sieved at a mean dimension lower than 355 μm.

### 2.3. Loading Tests

**Figure 1:**
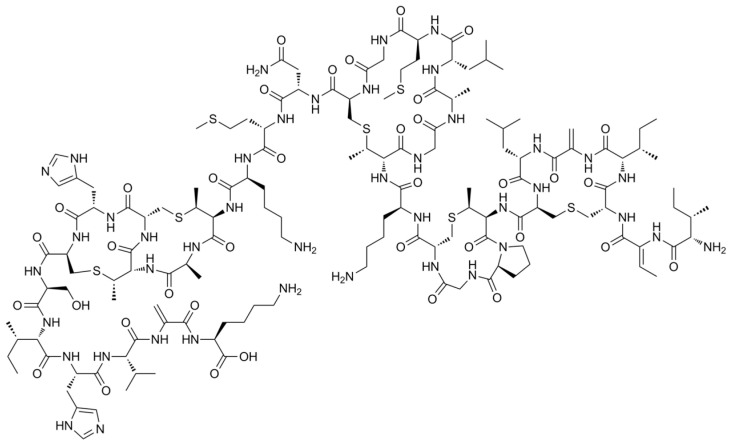
Nisin structure.

Loading tests were conducted choosing a molecule able to discriminate between LPMS and MS pores. For this reason, loadings were performed with Nisin (Figure 1) (>900 IU/mg, min 50% NaCl, Biosynth, Nobelova, Slovakia), an antimicrobial and biocompatible peptide that is 5.6 nm big [21,22,23].

Different loading solution concentrations were studied (from 0.5 to 20 mg of Nisin per mL of deionized water). Loading tests were performed by soaking 125 mg of the sample with 5 mL of loading solution, in a thermos-shaker at 25 °C, 120 rpm for 24 h; after that, solutions were centrifuged, and the solids were collected, washed and treated at 70 °C overnight to remove water.

On dried solids, we performed Elemental Analysis to evaluate the Nisin content; therefore, Nisin solutions were collected to spectrophotometrically determine the Nisin content before and after soaking. Spectrophotometric quantification was performed using a Nisin calibration curve in a concentration range of 0.2–1 mg/mL of Nisin in deionized water.

### 2.4. Nisin Release in Simulated Body Fluid (SBF)

Dried solids were used to conduct Nisin release tests just after solid drying and after a few weeks to evaluate Nisin stability [24]. Dried solids were preserved in anhydrous and dark conditions, and both release tests gave same results. First, 50 mg of sample were soaked with 5 mL of SBF solution for different timing (from 0.5 up to 96 h) in a thermos-shaker at 37 °C, 120 rpm; then, the suspension was centrifuged, and the supernatant was collected to conduct a spectrophotometric quantification of the Nisin released. Spectrophotometric measures were performed using a Nisin calibration curve in a concentration range of 0.05–0.25 mg/mL of Nisin in SBF. After quantification, a new amount of SBF was put in contact with the just-soaked solid, and so on until arriving to the total release of Nisin.

Experiments regarding Nisin stability in SBF were performed to confirm the presence of Nisin after a long period of soaking in SBF, using a reference solution. The time intervals tested were the ones considered for release tests, and a UV-Vis quantification of Nisin confirmed its stability.

### 2.5. Physical-Chemical Characterization of Powders

#### 2.5.1. Scanning Electron Microscopy (SEM)

The morphology of the unaltered powdery samples, of the loaded powdery samples and of samples after soaking in SBF was examined by SEM-FEG using a SEM Nova NanoSEM 450 (FEI Company, Milan, Italy) microscope operating at 15 kV.

#### 2.5.2. Textural Properties

To evaluate the loading behavior of LPMSs, the surface areas were determined by N_2_ adsorption/desorption isotherms carried out at T~77 K using ChemiSorb 2750—Micromeritics (Alfatest S.r.l., Rome, Italy). Adsorption data were processed by the standard Brunauer, Emmet and Teller (BET) method [25] to determine the specific surface area (SSA_BET_).

The total pore area and the intrusion volume were determined with a mercury porosimeter, using AutoPore IV 9500 (Micrometrics Instrument Corporation, Alfatest S.r.l., Rome, Italy) operating at mercury filling pressure of 1.51 psi.

The pore size can be determined from the pressure value of the liquid through the Washburn equation (Equation (1)), which describes its capillary flow within them.

(1) $$L=\sqrt{\frac{\gamma rt\cos ϴ}{2\eta }}$$

where *L* is the penetration length, *γ* is the surface tension, *r* is the pore radius, *θ* is the contact angle between the penetrating liquid and pore walls, *t* is the penetration time and *η* is the dynamic viscosity.

#### 2.5.3. UV-VIS Spectroscopy

To evaluate the amount of Nisin loaded inside the structures and the amount released after soaking with SBF, UV-VIS spectra were acquired. UV-visible spectra were recorded using a JASKO V-570 UV/Vis/NIR (JASCO Europe S.r.l., Cremella, Italy) (spectrophotometer at 298 K in the 190–400 nm spectral range employing quartz cells (1 cm optical path). Nisin quantification was performed at 277 nm.

#### 2.5.4. Elemental Analysis (EA)

EA was performed to confirm data collected with UV-VIS spectra, evaluate the amount of Nisin loaded inside the structures and evaluate its stability, using Thermo Scientific™ FLASH 2000 CHNS Anal (Thermo Fischer Scientific Inc., Milan, Italy).

A process blank was prepared and analyzed for all samples, and the results show this taken into consideration.

#### 2.5.5. Thermogravimetric Analysis (TG-DTA)

To confirm the presence of stable Nisin onto the structures, TG analyses were performed using a Seiko SSC 5200 in a temperature range between 25 °C and 1000 °C with a heating rate of 1 °C/min.

#### 2.5.6. Confocal Laser Scanning Microscopy (CLSM)

A confocal microscope was used to evaluate the presence of Nisin on the surface of powdery samples loaded with Nisin. Nisin fluorescence was obtained using an exciting wavelength of 405 nm and registering the emission in the range 470–580 nm, using Leica TCS SP8 (Leica Biosystems, Milan, Italy).

## 3. Results and Discussions

### 3.1. LPMSs’ Morphology Characterization with SEM

To choose the correct time of reaction and the quantity of adjuvant, SEM-FEG characterization of samples was performed.

**Figure 2:**
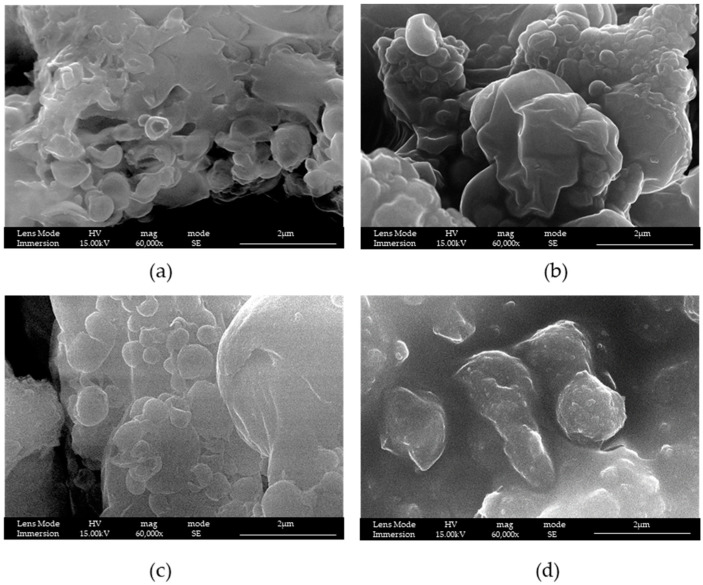
SEM-FEG images of samples (**a**) LPMS_1_TMB_18h, (**b**) LPMS_2_TMB_18h, (**c**) LPMS_1_18h and (**d**) LPMS_2_18h.

In this work, we started to study the product of the reaction by fixing the surfactant, and using the classical P123^®^ to synthesize MS [26,27]. In Figure 2, the samples differ in terms of the presence or not of the adjuvant and its amount: in LPMS_1_TMB_18h, the quantity of adjuvant is twice with respect to LPMS_2_TMB_18h, and the difference in the aperture of the structure is noticeable; in LPMS_1_18h and LPMS_2_18h, adjuvant is not present, and the structures are more compact. For these reasons, the presence of an adjuvant was considered necessary, and a ratio by mass 1:3.85 TMB:TEOS was fixed.

**Figure 3:**
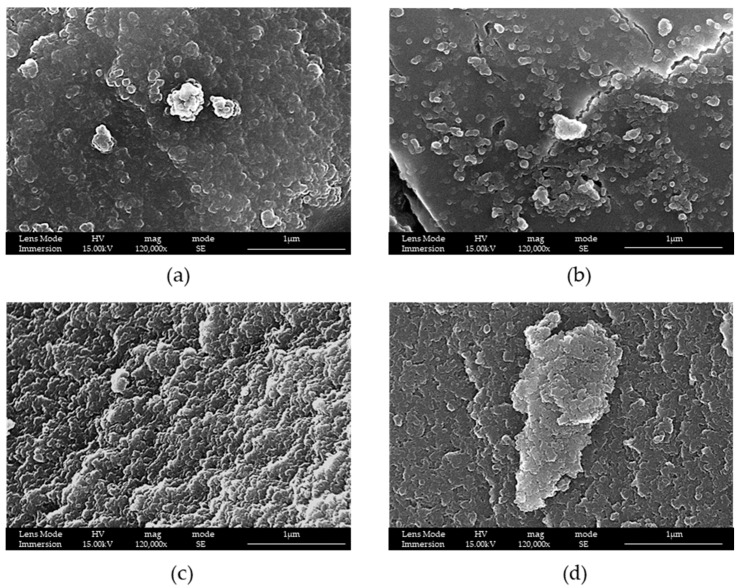
SEM-FEG images of samples (**a**) LPMS_1_2h, (**b**) LPMS_1_4h, (**c**) LPMS_1_6h and (**d**) LPMS_1_8h.

Then, maintaining the same ratio by mass of TEOS:surfactant at 1.7:1 and TEOS:TMB at 3.85:1 of the synthesis LPMS_1_TMB_18h, and varying only the time of reaction, we tried to reduce it. It is possible to notice from Figure 3, starting from 2 h and arriving up to 8 h, that the only result was that the surface became more and more non-homogeneous, but the best time remains at 18 h.

It is possible to notice from Figure 2 and Figure 3 that the increasing time brings a better aggregation of the silica nanoparticles and that, at 2 h, they are abundantly present compared to at 18 h, when more sintered structures are present.

After thatwe studied if it was possible to obtain better structures by fixing the time at 8 h, and modifying the ratio by mass TEOS:surfactant from 1:1.0 to 1:2.0.

**Figure 4:**
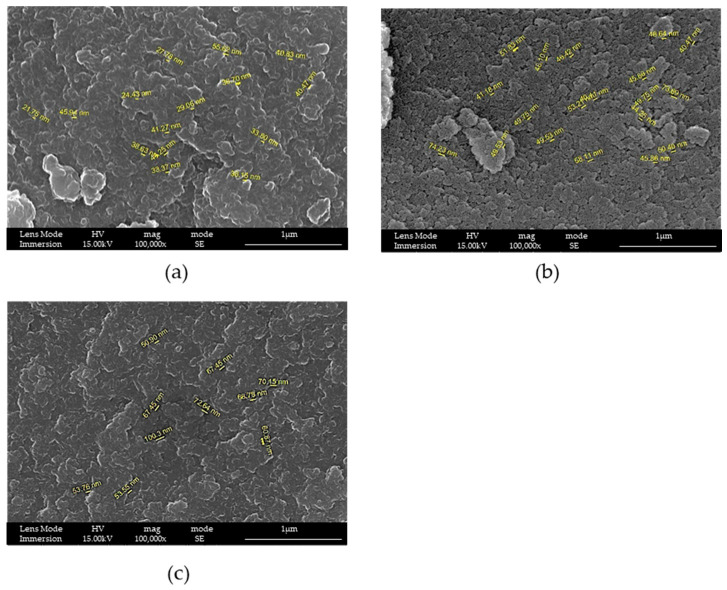
SEM-FEG images of samples (**a**) LPMS_3_8h ratio by mass 1:1, (**b**) LPMS_4_8h ratio by mass 1:1.4 and (**c**) LPMS_5_8h ratio by mass 1:2. SEM images report the cavity dimensions measured with specific SEM-FEG software tool.

It is possible to see from Figure 4 that an increase in the quantity of surfactant causes an increase in pores’ dimensions, which was the intended purpose. For this reason, the ratio by mass of TEOS:surfactant was fixed to 1:2.

It is clear that 18 h of MW treatment seems to be better to obtain the desired structure, but for studying reasons, we decide to also perform studies on samples obtained with 8 h thermal treatment.

At this point, a new surfactant was studied. Pluronic^®^ F127 was considered, thanks to its polyethyleneoxy chain that is five times longer with respect to P123 [28].

Taking into account the previous considerations, a new synthesis was identified: LPMS_7_TMB_18h in which the ratio by mass of TEOS:surfactant was fixed at 1:2 and the ratio by mass of TEOS:TMB was fixed to 3.85:1 with the time of reaction at 18 h.

**Figure 5:**
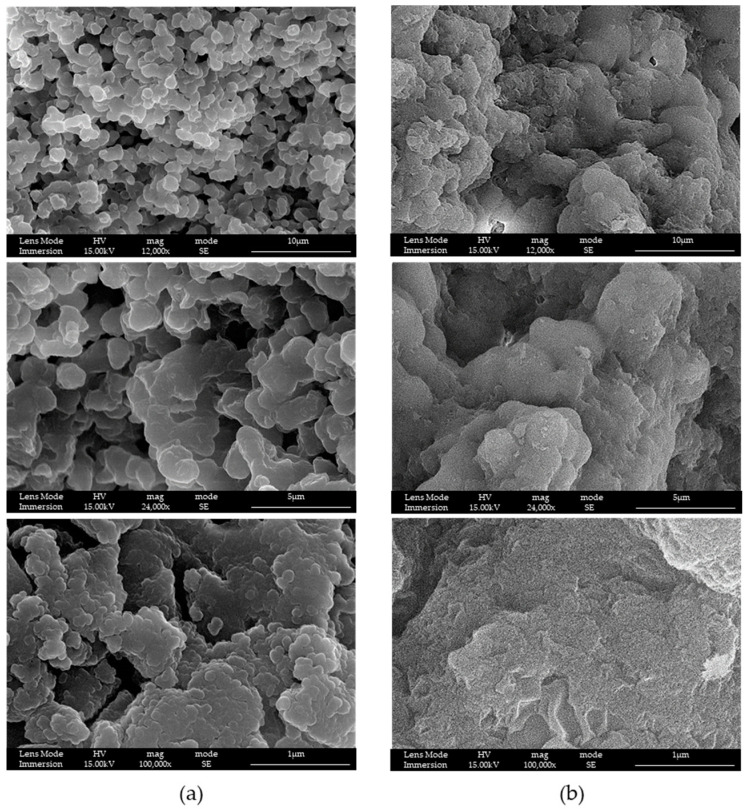
SEM-FEG images of (**a**) LPMS_7_TMB_18h and (**b**) LPMS_6_TMB_18h at different magnifications. The difference between samples stays in surfactant: LPMS_7_TMB_18h is synthesized using F127 and LPMS_6_TMB_18h using P123.

In Figure 5, is possible to evaluate the difference between LPMS_7_TMB_18h and an equivalent synthesis, LPMS_6_TMB_18h, in which the only difference stays in the surfactant used, which in the second case was P123.

With F127, a great enhancement in the morphology of the structure was obtained: silica nanoparticles were well-sintered and a more open structure and bigger mesopores were obtained. For these reasons, the second surfactant, F127, was the right candidate for our purposes and was fixed as the appropriate surfactant for the next steps.

**Figure 6:**
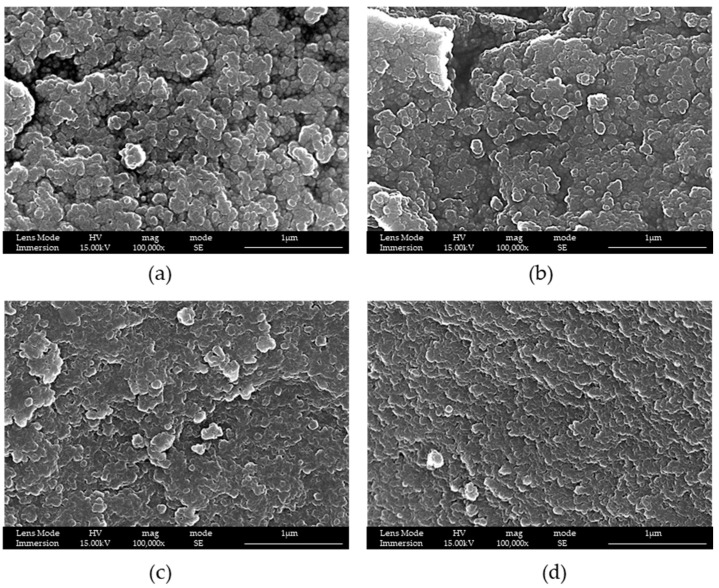
SEM-FEG images of samples (**a**) LPMS_8_TMB, (**b**) LPMS_9TMB, (**c**) LPMS_10_TMB and (**d**) LPMS_11_TMB. Ratios of TEOS:surfactant are respectively (**a**) 1:2, (**b**) 1:1.7, (**c**) 1:1.4 and (**d**) 1:1.0.

Additionally, with F127, reactions at 8 h and with different ratios by mass were studied, as reported in Figure 6.

Even with F127, if the ratio by mass between TEOS and the surfactant was changed, a more opened structure was obtained. Additionally, in this case, the ratio of 1:2 was the best.

At this time, as a preliminary analysis, an estimation of pores’ dimension [29] and a correlation between their dimensions and the quantity of surfactant was performed, considering the reaction conducted at 8 h with ratios by mass of 1:2.0, 1:1.7, 1:1.4 and 1:1.0. Both surfactants were studied, and the obtained results are reported in Table 2.

**Figure 7:**
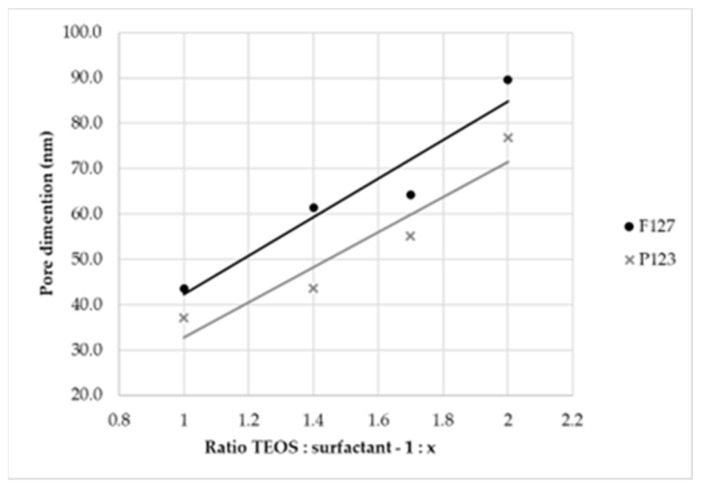
Graph of linear correlation between pores’ dimensions (calculated with the length tool of SEM-FEM software ESPRIT 2.1) and ratio of TEOS:surfactant used for the syntheses.

From the estimation, a linear relationship (Figure 7) between pores’ dimensions and the amount of surfactant used in the synthesis, i.e., between pores’ dimensions and the ratio by mass, was observed. Additionally, thanks to the bigger dimensions of F127 in terms of the carbon chain, larger pores were obtained with this surfactant [30].

For these reasons, to achieve our purposes, the optimal ratio by mass was 1:2.0, and F127 was the appropriate surfactant.

This determination was performed with exploratory purposes, and samples selected for the following studies were completely and properly characterized (see Section 2: Textural Properties).

To summarize all our findings: the presence of an adjuvant helps to obtain bigger pores, so TMB was necessary; the best structures in terms of compactness were obtained at a higher reaction time; therefore, samples obtained at 8 h and 18 h were considered in the next steps; the best surfactant is the biggest one; consequently, F127 was chosen; at a higher ratio by mass, bigger pores were obtained, and a ratio of 1:2 of TEOS:surfactant was fixed.

Taking into account all these considerations, the samples studied in the next steps will be LPMS_8_TMB_8h and LPMS_7_TMB_18h.

**Figure 8:**
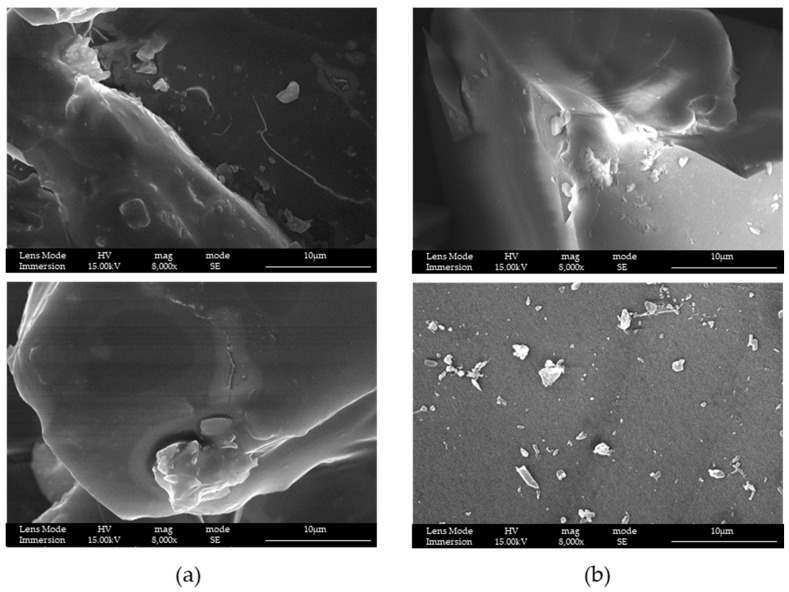
SEM-FEG images of samples (**a**) MS and (**b**) WMS at different magnifications.

SEM-FEG characterization (Figure 8) was also performed for structures used as a reference in loading and release tests. Reference structures considered were MS and silica with no mesopores, only containing micropores (without mesoporous silica, WMS).

It is possible to see, that both structures look compact if compared with the same magnification of LPMS_8_TMB_8h or LPMS_7_TMB_18h, respectively, reported in Figure 4 and Figure 5.

### 3.2. Textural Properties

**Figure 9:**
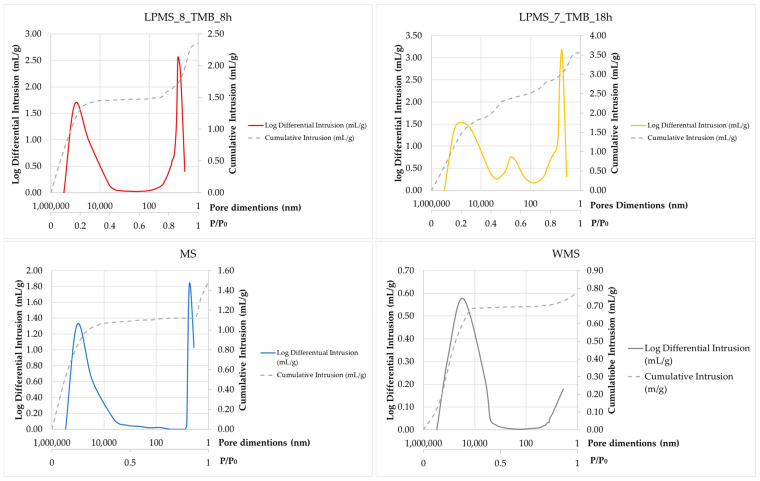
Adsorption/desorption isotherms based on which the surface area values were obtained. From cumulative intrusion (dashed line), it is possible to notice a different behavior between MS and LPMS.

N_2_ adsorption/desorption and mercury impregnation measurements were performed to evaluate and compare the textural properties of samples, determining the following: specific surface area (SSA_BET_), total pore area (A_p_) and total intrusion volume (V_p_). The first parameter was determined with N_2_ adsorption/desorption method as a mean of three different and independent measurements, and the others with mercury impregnation. In Table 3, the textural parameters of samples are reported. Adsorption/desorption isotherms based on which the surface area values were obtained are presented in Figure 9. As it is possible to see from Figure 9, a different behavior between MS and LPMSs is evident: from the dashed lines, the intrusion in pores of big dimensions for LPMSs is noticeable, which is not detectable for MS and WMS.

SSA_BET_ was not significantly different between samples considered. WMS shows the highest value, due to the presence of micropores that significantly enhance this value. LPMS_8_TMB_8h and LPMS_7_TMB_18h show a value of SSA_BET_ that is consistent with the values obtained for classical MS [31]. This behavior can be justified by also considering the other values (A_p_ and V_p_), which are significantly higher for LPMSs with respect to the other structures [32].

A_p_ has the lowest values for WMS, which in line with the results considering that the sample only has micropores and the porosimeter cannot measure their pore area; MS, SSA_BET_ and A_p_ are comparable, instead of LPMSs that show an SSA_BET_ lower than the A_p_. It can be explained considering that SSA_BET_ was calculated using N_2_ adsorption/desorption and that Ap uses Hg; the latter can fill big pores, instead of N_2_, which can only create a layer on the surface of them, so it turns out that A_p_ can have higher values if compared to SSA_BET_ [33].

**Figure 10:**
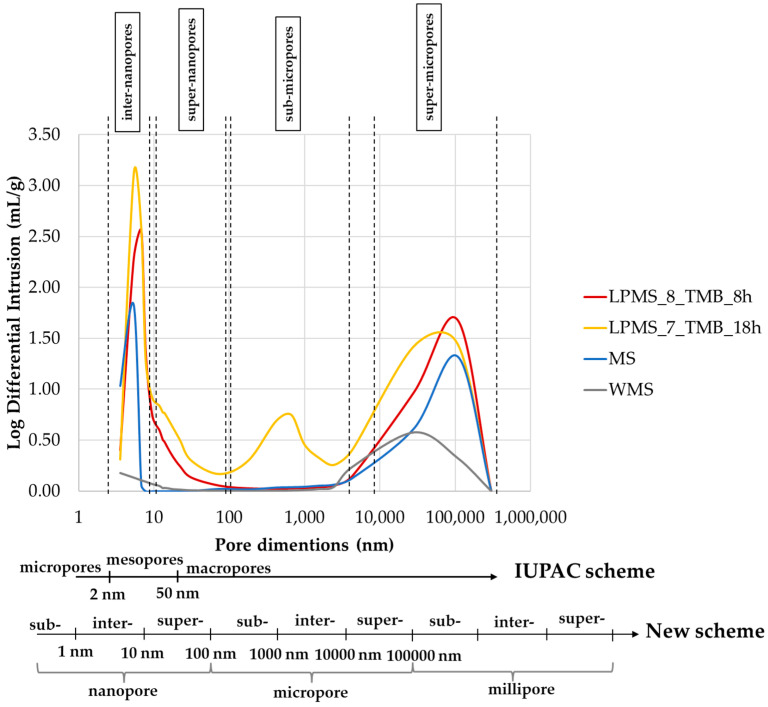
On the top: results obtained with the mercury porosimeter, and a graph of the distribution of pore volume as a function of pore size. In the bottom: pore classification due to dimension according to the IUPAC scheme and a new scheme proposed by T. J. Mays et al. [35].

The mercury impregnation method can determine pores only in the range of 3.5–300 µm. Figure 10 reports results obtained with the mercury porosimeter, and the classification of pores due to dimensions according to the IUPAC scheme [34] and a new scheme proposed by T. J. Mays, et al. [35]. According to particle dimensions, super-micropores can be considered interparticle pores. Pores under 10 µm can be classified, in order of dimensions, as follows: inter-nanopores (1–10 nm), super-nanopores (10–100 nm) and sub-micropores (100–1000 nm). Samples studied in this work present different characteristics in terms of pores, and the type and number of pores present in their structure are summarized in Table 4.

### 3.3. Nisin Loading Tests

The efficiency of the loading of a molecule into a structure can be calculated in different ways, using the following: loading efficiency percentage (LE%), loading percentage (Loading%) or loading capacity percentage (LC%).

The loading efficiency percentage (Equation (2)) considers the only amount of Nisin loaded inside the structure as a function of the initial quantity used for loading [36]:

(2) $$\mathrm{LE}\%=\frac{Amount\;of\;Nisin\;loaded\;\left(mg\right)}{Initial\;amount\;of\;Nisin\;\left(mg\right)}\cdot 100$$

The initial amount of Nisin was determined directly using a spectrophotometric quantification of the concentration of the loading solution; then, the amount of Nisin loaded can be determined as the difference between the initial concentration and the concentration after loading, which is always determined by spectrophotometric measure.

The loading percentage (Equation (3)) considers the amount of Nisin loaded as a function of the total weight of the loaded structure [37]:

(3) $$\mathrm{Loading}\%=\frac{Amount\;of\;Nisin\;loaded\;\left(mg\right)}{Weight\;of\;loaded\;structure\;\left(silica+Nisin\right)\;\left(mg\right)}\cdot 100$$

Loading% can be also calculated, to compare results, using the EA results as follows (Equation (4)):

(4) $$\mathrm{LE}\%=\frac{\%\;C\;determined\;with\;EA}{Theorethical\;\%C}\cdot 100$$

Finally, the loading capacity percentage was calculated as a function of the amount of Nisin per milligrams of silica (Equation (5)) [38,39]:

(5) $$\mathrm{LC}\%=\frac{Amount\;of\;Nisin\;loaded\;\left(mg\right)}{Silica\;amount\;\left(mg\right)}\cdot 100$$

EA was performed on solids collected after loading and dried overnight. In Table 5, the EA and samples are reported and were labeled as “sample_name_x” in which x indicates the concentration of Nisin (in mg/mL) in the loading solution.

EA results were used to calculate loading% and to evaluate the stability of Nisin loaded into the structures. As it is possible to see from Table 4 that the results are in accordance with the concentration of the loading solution: the higher the concentration, the higher the percentage of each element in the structures.

**Figure 11:**
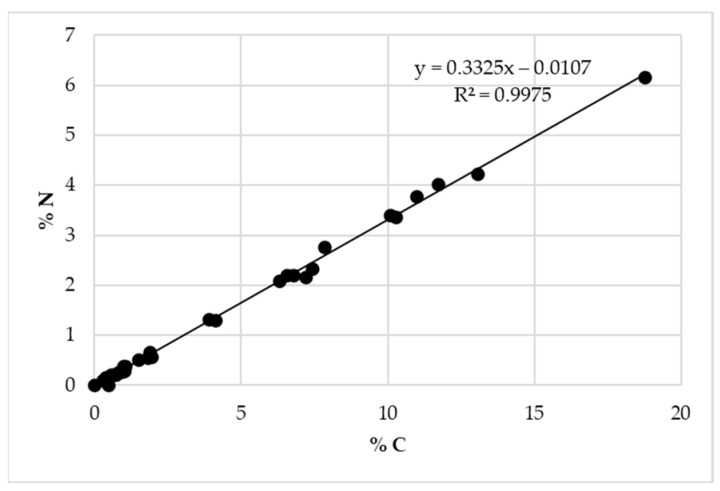
Linear regression was obtained by plotting %N depending on the %C obtained from EA. In the graph, all studied and loaded samples are reported.

To evaluate the presence of Nisin on the surface of the structures, %N vs. %C obtained with EA were plotted for all samples (Figure 11), and the slope of linear regression obtained was compared to the ratio %N/%C calculated for pure Nisin. Considering the Nisin molecular formula (C_143_H_230_N_42_O_37_S_7_), a %N/%C of 0.343 is obtained, which is perfectly in line with the result if compared to the 0.333 obtained with experimental linear regression. For this reason, it can be confirmed that the loaded Nisin is still present, but to assert the presence of non-degraded Nisin and also confirm its presence in the structures after various weeks, a UV-Vis determination is necessary. This aspect will be treated in Section 4: Results and Discussion.

**Figure 12:**
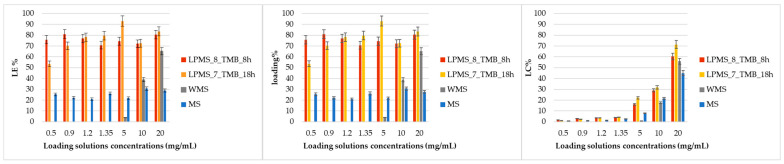
LE%, loading% and LC% of all samples reported in Table 5 are graphed in a bar graph to better understand and compare data collected for LPMSs, MS and WMS.

Comparing LE%, LC% and loading% reported in Table 6 and in Figure 12, it could be noticed that the LE% for LPMS_8_TMB_8h and LPMS_7_TMB_18h does not vary significantly at the different loading concentrations and always stays in a range between 50 and 90%, instead of MS, which shows a LE% in the range 20–30%, which is significantly lower than LPMSs; WMS has a LE% that varies exponentially, synonymous with a process under precipitation control. The same results were obtained for Loading%. These effects are caused by the dimensions of pores: for LPMSs, Nisin can fill very large cavities, and the trapped amount is up to 3/4 times higher with respect to classical structures; in MS and WMS, Nisin can stay only on the surface, and the Nisin amount detected is the Nisin precipitated on the surface. This way of loading will bring very fast and not controlled releases, if compared to LPMSs. LC% has, for all samples, an exponential behavior because the more Nisin in the solution, there is more quantity that can be trapped or that can recover the surface.

The Loading% values calculated with EA results are consistent with the ones calculated spectrophotometrically.

TGA-DTA was performed on structures with a comparable LC%, to obtain a decomposition of Nisin in the same mass range, i.e., LPMS_8_TMB_8h_1.35, LPMS_7_TMB_18h_1.35, MS_1.35 and WMS_5.

**Figure 13:**
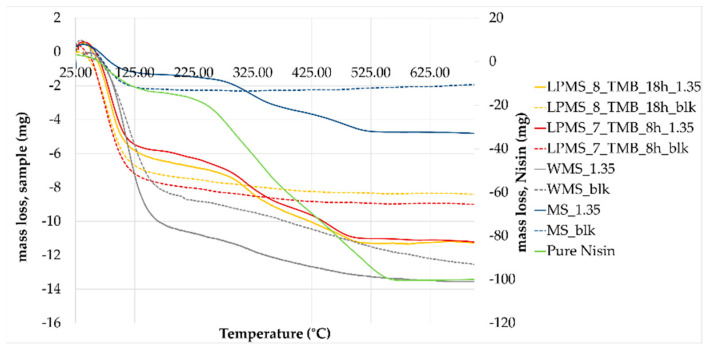
TG graph of loaded samples (full lines) and unprocessed samples (dashed lines). From the differences between curves, it is possible to calculate the loss in mass related to the Nisin content.

**Figure 14:**
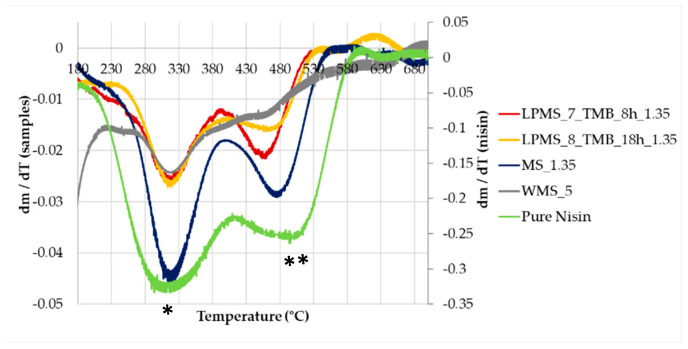
DTG, derivative graph obtained from TG curves. *, peak relative to pyrolisis of Nisin; **, peak relative to the decomposition of Nisin.

TGA (Figure 13) confirmed the results obtained with the previous analysis, and the loss in mass obtained with thermal analysis is comparable to the amount of Nisin established with EA and UV-VIS determination (Table 7). To determine the loss in mass of loaded structures, unprocessed samples were also analyzed as a blank reference. From the TG derivative graph (DTG) (Figure 14), a delay in decomposition, with respect to pure Nisin, was observed for all samples, probably caused by the interaction between Nisin and the silica surface. The same derivative shapes of a sample of pure Nisin were obtained, confirming the presence and stability of Nisin loaded onto the structures. In the derivative graph, the first peak at 300 °C is attributable to the pyrolysis of Nisin (*), and the second one (**) at about 480 °C is attributable to the decomposition of the peptide structure [40].

**Figure 15:**
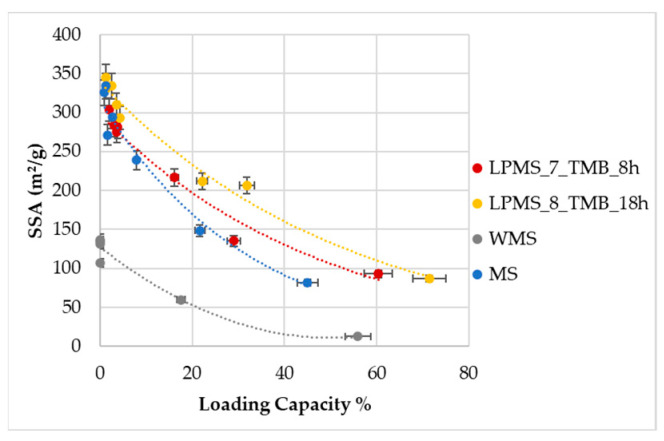
SSA_BET_ determined on unadulterated powders and on loaded powders, plotted as a function of LC%. Data are reported as a mean of three different measures performed on three different amounts of sample.

BET was determined for all loaded samples to evaluate the behavior of LPMS and MS. From SSA_BET_ values plotted as a function of LC% (Figure 15), it is possible to see that LPMSs show a lower decrease in SSA if compared to MS; considering the difference in LE% between samples, this behavior could be explained considering first the filling of pores and then, at high concentration, the covering of the surface for LPMSs that permit the slowest decrease of SSA_BET_, instead of MS, which undergoes a pore blocking [41,42], which is well-known for mesoporous materials. Additionally, superficial analysis confirms the different behavior between WMS and other samples.

### 3.4. Nisin Release Tests

To evaluate Nisin stability, Nisin release tests were performed on loaded structures a few weeks after loadings. The Nisin content obtained spectrophotometrically and with EA is comparable, so can be asserted that Nisin is present and stable.

Nisin release tests were performed for structures with a comparable Loading%, i.e., LPMS_8_TMB_8h_5, LPMS_7_TMB_18h_5, MS_10 and WMS_10, which all have a Loading% of about 15%.

The amount of Nisin released was determined as % of release (Equation (5)):

(6) $$\%\;\mathrm{release}=\frac{Cumulative\;amount\;of\;Nisin\;released\;at\;a\;certain\;time}{Total\;Nisin\;content}\cdot 100$$

**Figure 16:**
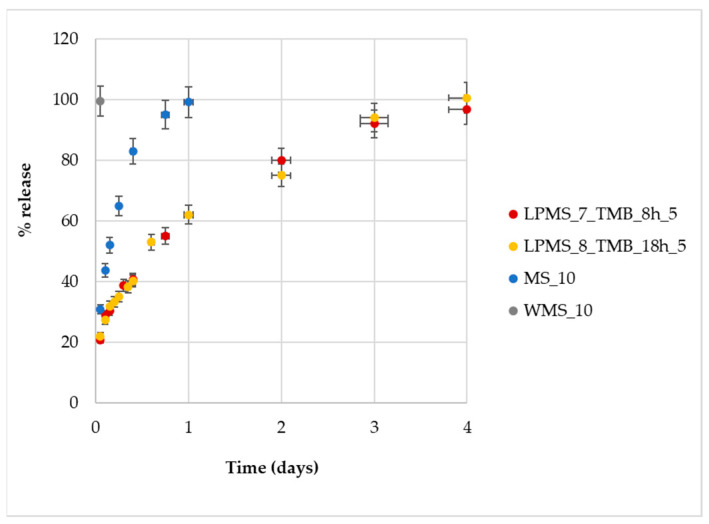
%Release determined with UV-Vis analysis plotted as a function of time.

Starting from samples with the same Nisin content, a four time longer release and controlled mechanism of release can be achieved for LPMSs (Figure 16). MS exhibits a shorter release, and even shorter for WMS, of about 1 day and 1 h, respectively, in comparison to 4 days of LPMSs. LPMSs’ releases are definitely longer if compared with the time of the release of MS and previous studies [43]: LPMSs show releases that last 96 h, instead of MS, which shows a four time shorter release of about 24 h (results in line with previous studies).

**Figure 17:**
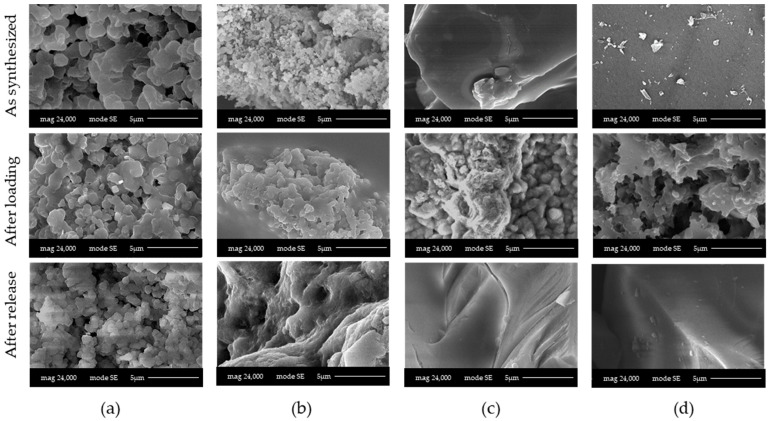
SEM-FEG images acquired on samples (**a**) LPMS_7_TMB_18h_5, (**b**) LPMS_8_TMB_8h_5, (**c**) MS_10 and (**d**) WMS_10, studied unaltered, after loading and after release tests in SBF.

Another important aspect is the mechanical resistance that LPMS_7_TMB_18h shows after being kept in contact with SBF for a lot of hours. In Figure 17, we report the SEM-FEG images of unaltered structures, loaded structures and structures after soaking in SBF.

After release, LPMS_7_TMB_18h maintains its structure, synonymous with mechanical resistance; LPMS_8_TMB_8h does not have the same mechanical resistance, synonymous with the fact that a longer thermal treatment is necessary to obtain structures that can resist over time when used as support for drug release. For MS and WMS, after a release of 24 h or 1 h, respectively, the structures are maintained.

**Figure 18:**
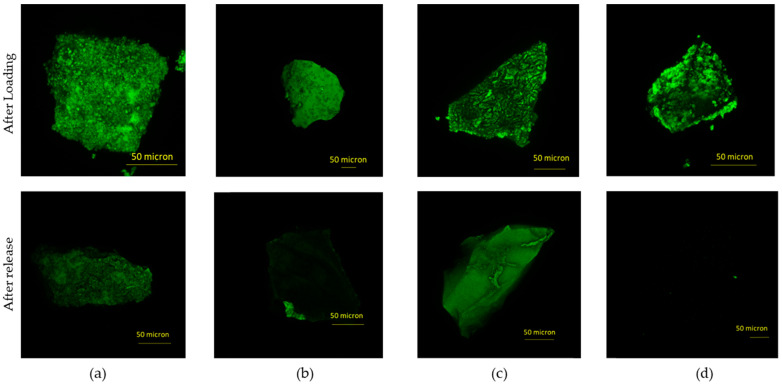
Confocal laser scanning microscopy images acquired on samples (**a**) LPMS_7_TMB_18h_5, (**b**) LPMS_8_TMB_8h_5, (**c**) MS_10 and (**d**) WMS_10 studied when loaded and after release in SBF.

CLSM was used to visibly evaluate Nisin loading. In Figure 18, microscope images obtained for loaded samples and samples after release are reported.

As it is possible to see from a comparison between the first and the second row in Figure 18, the confocal microscope images confirm the same patterns observed at SEM-FEG: the structure of LPMS_8_TMB_18h is also maintained after loading and the pores are visibly filled with Nisin, LPMS_7_TMB_8h after loading has a more compact structure due to the filling of superficial cavities and MS and WMS pass from a smooth surface to a non-homogeneous surface due to Nisin precipitation on the surface of the microparticles.

The hypothesis of an in-pore drug loading for LPMSs and an external deposition of Nisin for MS and WMS was confirmed.

In fact, loaded structures of MS and WMS show a change in the superficial aspect, indicating that Nisin precipitates on the surface, and for LPMSs, it is noticeable for the filling of pores.

In addition, complete releases can be demonstrated. As it is possible to see from images, the fluorescence of samples is highly quenched, synonymous with an almost complete release.

## 4. Conclusions, Limitations and Prospects

In light of the results obtained, it can be concluded that a new synthesis pathway to obtain LPMSs has been drawn, and this study, concerning a better surfactant and reaction time, shows that a different methodology with respect to the old one has to be adopted, in terms of the surfactant, time, temperature and conditions of reaction.

In loading and release tests, LPMSs show improved characteristics with respect to the old structures, in terms of LE%, LC%, controlled and longer release and mechanical resistance in physiological environment.

In conclusion, LPMSs (in particular, LPMS_7_TMB_18h) are good candidates for reaching high loading and controlled drug release of big pharmaceutical molecules, thanks to the structured pore system and mechanical resistance. Their utilization in studying the mobility of large organic molecules could be considered in a confined state [44]. However, the weak point of synthesized structures could be the superficial pores’ blocking act by substances that can be found in vivo, such as cells.

## Figures and Tables

**Table 1:** Synthesis conducted.

Sample	Type of Surfactant	Reaction Time (h)	Mass Ratio TEOS/Surfactant	Surfactant (g)	TEOS (g)	TMB (mL)	HCl (1.7%) *w*/*w* (mL)
LPMS_1_TMB_18h	P123	18	1.7	3.4	2.0	0.52	140
LPMS_2_TMB_18h	P123	18	1.7	3.4	2.0	0.26	140
LPMS_1_18h	P123	18	1.7	3.4	2.0	-	140
LPMS_2_18h	P123	18	1.7	3.4	2.0	-	140
LPMS_1_TMB_2h	P123	2	1.7	3.4	2.0	0.52	140
LPMS_1_TMB_4h	P123	4	1.7	3.4	2.0	0.52	140
LPMS_1_TMB_6h	P123	6	1.7	3.4	2.0	0.52	140
LPMS_1_TMB_8h	P123	8	1.7	3.4	2.0	0.52	140
LPMS_3_TMB_8h	P123	8	1.0	2.0	2.0	0.52	140
LPMS_4_TMB_8h	P123	8	1.4	2.8	2.0	0.52	140
LPMS_5_TMB_8h	P123	8	2.0	4.0	2.0	0.52	140
LPMS_6_TMB_18h	P123	18	2.0	4.0	2.0	0.52	140
LPMS_7_TMB_18h	F127	18	2.0	4.0	2.0	0.52	140
LPMS_8_TMB_8h	F127	8	2.0	4.0	2.0	0.52	140
LPMS_9_TMB_8h	F127	8	1.7	3.4	2.0	0.52	140
LPMS_10_TMB_8h	F127	8	1.4	2.0	2.0	0.52	140
LPMS_11_TMB_8h	F127	8	1.0	2.8	2.0	0.52	140

**Table 2:** Pores’ dimensions obtained for samples.

	Pores Dimensions ^1^ (nm)	
m TEOS:m Surfactant	Pluronic^®^ F172	Pluronic^®^ P123
**1:2.0**	89.7	76.8
**1:1.7**	64.2	55.1
**1:1.4**	61.3	43.6
**1:1.0**	43.5	37.1

^1^ Pores’ dimensions calculated as the average of 30 measures collected with the length tool of SEM-FEG software.

**Table 3:** SSA_BET_, A_P_ and V_p_ of samples subjected to loading and release tests.

Sample	SSA_BET_ (m^2^/g)	A_P_ (m^2^/g)	V_p_ (mL/g)
LPMS_8_TMB_8h	309 ± 15	421	2.36
LPMS_7_TMB_18h	330 ± 17	499	3.57
MS	323 ± 16	289	1.49
WMS	404 ± 21	50	0.78

**Table 4:** Type and number of pores of samples at a certain range of dimensions.

	3–10 nm	10–30 nm	500–600 nm	10–200 µm
Sample	Inter-Pore	Super-Nanopore	Sub-Micropore	Super-Micropore
LPMS_8_TMB_8h	++++	++	-	++++
LPMS_7_TMB_18h	+++++	+++	+++	++++
MS	+++	-	-	++++
WMS	+	-	-	++

The number of “+” schematically indicates the number of pores at a certain dimension.

**Table 5:** Results of EA performed on loaded structures dried overnight at 70 °C.

Sample	%C	%H	%N	%S
LPMS_8_TMB_8h_0.5	0.78	0.36	0.25	0.00
LPMS_8_TMB_8h_1	0.80	0.31	0.26	0.00
LPMS_8_TMB_8h_1.2	1.88	0.58	0.67	0.08
LPMS_8_TMB_8h_1.35	1.96	0.50	0.58	0.00
LPMS_8_TMB_8h_5	7.21	1.30	2.17	0.00
LPMS_8_TMB_8h_10	10.27	1.99	3.36	0.50
LPMS_8_TMB_8h_20	13.05	1.85	4.24	0.64
LPMS_7_TMB_18h_0.5	0.58	0.23	0.22	0.00
LPMS_7_TMB_18h_1	0.97	0.30	0.39	0.00
LPMS_7_TMB_18h_1.2	1.49	0.36	0.52	0.00
LPMS_7_TMB_18h_1.35	1.83	0.48	0.55	0.08
LPMS_7_TMB_18h_5	6.56	1.09	2.20	0.32
LPMS_7_TMB_18h_10	10.99	1.35	3.79	0.00
LPMS_7_TMB_18h_20	11.70	1.60	4.02	0.20
MS_0.5	0.51	0.08	0.15	0.00
MS_1	0.73	0.10	0.22	0.00
MS_1.2	0.92	0.13	0.28	0.00
MS_1.35	1.01	0.15	0.30	0.00
MS_5	4.12	0.83	1.30	0.06
MS_10	6.77	0.89	2.21	0.05
WMS_0.5	0.29	0.32	0.11	0.00
WMS_1	0.39	0.69	0.15	0.00
WMS_1.2	0.54	0.00	0.20	0.00
WMS_1.35	0.46	0.08	0.00	0.00
WMS_5	1.06	0.61	0.38	0.00
WMS_10	7.85	1.14	2.77	0.24
WMS_20	10.09	1.70	3.41	0.43

**Table 6:** LE%, LC%, loading% and loading% calculated with EA of all samples studied.

Sample	LE%	LC%	Loading%	Loading% Calculated with EA
LPMS_8_TMB_8h_0.5	75.8	1.9	1.8	1.7
LPMS_8_TMB_8h_1	81.0	3.1	3.0	1.7
LPMS_8_TMB_8h_1.2	76.9	3.6	3.5	3.8
LPMS_8_TMB_8h_1.35	70.7	3.8	3.7	4.0
LPMS_8_TMB_8h_5	74.5	16.1	13.8	14.2
LPMS_8_TMB_8h_10	72.1	29.0	26.6	20.2
LPMS_8_TMB_8h_20	80.5	60.4	43.0	25.6
LPMS_7_TMB_18h_0.5	53.6	1.2	1.2	1.3
LPMS_7_TMB_18h_1	70.2	2.5	2.5	2.0
LPMS_7_TMB_18h_1.2	78.2	3.5	3.4	3.0
LPMS_7_TMB_18h_1.35	79.7	4.3	4.1	3.7
LPMS_7_TMB_18h_5	93.1	22.1	18.1	12.9
LPMS_7_TMB_18h_10	72.6	31.8	24.1	21.6
LPMS_7_TMB_18h_20	83.5	71.4	41.7	23.0
MS_0.5	25.3	0.9	0.9	1.0
MS_1	22.2	1.2	1.2	1.5
MS_1.2	21.0	1.6	1.5	1.8
MS_1.35	26.2	2.6	2.5	2.0
MS_5	21.8	7.9	7.3	8.1
MS_10	30.7	21.6	17.8	13.3
MS_20	29.0	45.0	27.6	26.8
WMS_0.5	0.0	0.0	0.0	0.6
WMS_1	0.0	0.0	0.0	0.8
WMS_1.2	0.0	0.0	0.0	1.1
WMS_1.35	0.0	0.0	0.0	0.9
WMS_5	3.8	0.9	0.9	2.1
WMS_10	38.9	17.6	15.0	15.4
WMS_20	65.3	55.9	35.8	19.7

**Table 7:** Total amount of Nisin (calculated as LC%) inside structures determined with different analyses.

	Nisin Content—Different Determination Techniques	
Sample	UV-Vis	TG
LPMS_8_TMB_8h_1.35	3.8 mg/100 mg	4.2 mg/100 mg
LPMS_7_TMB_18h_1.35	4.3 mg/100 mg	4.7 mg/100 mg
MS_1.35	2.6 mg/100 mg	2.8 mg/100 mg
WMS_5	0.9 mg/100 mg	1.2 mg/100 mg

## Data Availability

Additional data that support the findings of this study are available from the corresponding author.
